# General psychiatric or depressive symptoms were not predictive for mortality in a healthy elderly cohort in Southern Brazil

**DOI:** 10.1590/S1980-57642009DN20200008

**Published:** 2008

**Authors:** Maria Otilia Cerveira, Adelar Pedro Franz, Ana Luiza Camozzato, Márcia Lorena Fagundes Chaves

**Affiliations:** Dementia Clinic, Neurology Service, Hospital de Clínicas de Porto Alegre. Medical Sciences Post-Graduation Course, UFRGS School of Medicine. Internal Medicine Department, UFRGS School of Medicine

**Keywords:** psychiatric symptoms, depressive symptoms, mortality, elderly cohort, Brazil

## Abstract

General psychiatric symptoms may interfere with the ability of individuals to take care of their health, to get involved with activities and develop social abilities, thereby increasing risk of death.

**Objective:**

To evaluate general psychiatric symptoms as predictive factors for mortality in a community elderly cohort in Southern Brazil.

**Methods:**

345 healthy elderly, aged .60 years, from the catchment area of Hospital de Clinicas de Porto Alegre were followed from 1996. Data for the present study were drawn from the period 1996-2004. General psychiatric symptoms (Self-Reporting Questionnaire . SRQ), depressive symptoms (Montgomery-Asberg depressive rating scale), and Mini Mental State Examination scores at baseline were included in the study. Socio-demographic, medical conditions, and functional capacity were also analyzed. The outcome was vital status at follow-up obtained from family members, hospital records and checked against official death registers.

**Results:**

Of the 345 baseline individuals, 246 were followed-up. The global mortality rate over the study period was 36.9% (N=90). Those who deceased during the period were older (73.5±7.5), more dependent overall, and more cognitively impaired than the living elderly (univariate analyses). In the logistic regression, only age (OR=0.93; p=0.003) and functional capacity (OR=0.22; p=0.007) remained significant in the final equation.

**Conclusion:**

Psychiatric symptoms presented no association with mortality in the present sample. Older age and functional incapacity were risk factors for mortality.

## Abstract (pt)

Sintomas psiquiátricos gerais podem interferir com a capacidade do indivíduo cuidar de sua saúde, de envolver-se com outras atividades e desenvolver habilidades sociais, aumentando o risco de morte.

**Objetivo:**

Avaliar se os sintomas psiquiátricos são preditores de mortalidade em uma coorte de idosos de uma comunidade do sul do Brasil.

**Métodos:**

345 idosos saudáveis, com idade igual ou superior a 60 anos, da área de abrangência de um hospital universitário de uma cidade do sul do Brasil foram acompanhados desde 1996. Os dados desse estudo representam o período de avaliação de 1996 a 2004. Os sintomas psiquiátricos na avaliação inicial foram medidos através do *Self-Reporting Questionnaire* (SRQ) e da Escala Montgomery-Asberg para sintomas depressivos (MADRS) e as variáveis independentes foram desempenho cognitivo no Mini Exame do Estado Mental (MEEM). Dados sócio-demográficos, questionário sobre condições sociais e de saúde, e capacidade funcional também foram analizados. O desfecho principal foi mortalidade durante o seguimento, obtida através de familiares e registros de óbito.

**Resultados:**

Dos 345 idosos "baseline", dados foram obtidos de 246 no seguimento. Taxa de mortalidade global no seguimento foi de 36.9% (N=90). Aqueles que faleceram durante o período de avaliação eram mais idosos (73,5±7,5), mais globalmente dependentes e comprometidos do ponto de vista cognitivo do que os idosos que permaneceram vivos (análises univariadas). Na regressão logística, apenas idade (RC=0,93; p=0,003) e capacidade funcional (RC=0,22; p=0,007) mantiveram significância na equação final.

**Conclusões:**

Sintomas psiquiátricos não mostraram efeito preditor para mortalidade na presente amostra. Maior idade e incapacitação foram fatores de risco para mortalidade.

The growing impact of an aging population and the burden of non-communicable diseases call for a worldwide perspective together with a focus on the developing world. By 2025, around 70% of older citizens in the world will be living in these regions.

In the elderly population, cognitive impairment is associated with poorer survival compared with normal cognition.^1-3^ However, subjects with cognitive impairment are usually older, and their health is poorer than that of their cognitively unimpaired peers,^1-4^ leading to the hypothesis that age and diseases (such as depression, vascular diseases, diabetes, or physical comorbidity in general) rather than cognitive impairment per se might be responsible for the differential mortality.^5-7^ Depressive symptoms are important predictive factors of mortality when cognitive function is impaired.^8^

In a cohort with 9 years of follow-up, data evidenced the effect of several psychiatric problems (i.e., major depression, alcohol abuse or dependence, and schizophrenia) by demonstrating increased likelihood of mortality.^9^ A systematic review of the literature on depression, depressive symptoms and mortality in persons aged 65 and over living in the community showed that diagnosed depression was associated with increased mortality.^10^ On the other hand, psychological indices were not related to mortality risk^11^ and symptoms of depression were not associated with mortality in a cohort of older adults.^12^

The main objective of this study was to evaluate the association of general psychiatric and depressive symptoms with mortality in an elderly cohort in Southern Brazil.

## Methods

In 1996, 1,216 individuals out of 5,500 elderly aged 60 years and older who were residing in the catchment area of Hospital de Clinicas de Porto Alegre (Rio Grande do Sul, Brazil) according to data from the 1992 census,^13^ were enrolled in two studies. Of the 1,216 elderly, 848 presented cognitive deficit and/or major medical disorders and composed the sample for a further study. For this further study, 368 individuals were selected 23 of whom (6.3%) declined to participate. The total sample at baseline study was composed of 345 participants who fulfilled criteria for the study of healthy aging. The baseline study was conducted in two assessments with a short interval. The first was composed of selection and exclusion instruments, and the second of measures of interest. Each participant underwent a standardized neuropsychological and neurological evaluation. A collateral informant was also used to verify the history. Subjects were excluded if they had age-related diseases or presented risk factors for cognitive impairment at baseline. The exclusion criteria included medical conditions such as chronic renal disease, significant head injury, and stroke; psychiatric conditions such as major affective disorder or evidence of current depression; uncorrectable vision or hearing loss; or other conditions such as substance abuse or use of medications that might impair cognitive functioning. All participants and their collateral informants had to report normal functioning in the community at entry of study. To minimize inclusion of participants with incipient dementia, subjects were screened with the Clinical Dementia Rating scale.^14-16^ Participants with a CDR of 0.5 (suggestive of incipient dementia) or greater (suggestive of dementia) were excluded from the sample. The second interview collected detailed on demographic and medical information, social support, engagement in leisure activities, and included a scale to rate symptoms of depression (Montgomery-Asberg Rating Depression Scale),^17,18^ a questionnaire for general psychiatric symptoms (WHO Self-Report Questionnaire)^19,20^ the DSM IV criteria for Major Depression - MD, and the Mini-Mental State Examination.^21,22^ Independence for daily living activities was assessed with the ADL scale.^23^

Subjects for this study were participants in the ongoing longitudinal cohort study. Data for the present study were derived from the follow-up of up to 8 years. The statistical analysis was based on the diagnosis established at the last follow-up visit during which the participant underwent a thorough evaluation. At least one follow-up was completed for each of the two-hundred and forty-five participants.

For the present study, vital status (main outcome) was checked for the 246 participants during the 8-year follow-up, which corresponds to 71.3% of the baseline sample, and was representative of that group (Table 1). Vital status information was obtained from family members, hospital records and checked in the official registers of death. Among the deceased during the follow-up, retrospective data were obtained through a structured telephone interview with a knowledgeable collateral source focusing on dementia.^24,25^ The AD^8^ was additionally applied during the same telephone interview.^26^ Of the 246 participants, 90 had deceased during this period.

**Table 1:** Baseline population: age, sex, education, MMSE, family income, SRQ and MADRS at study entry (all and at least one follow-up completed).

Variables	All (N=345)	At least one follow-up completed (N=245)	P value
Age (mean±SD)*	70.37±7.15	70.87±6.86	0.36
Sex**			
Male (N, %)	103 (30%)	76 (31%)	0.79
Marital status**(living with partner)	159 (46%)	113 (46%)	1.0
MMSE (mean±SD)*	25.3±3.9a	25.4±3.6b	0.85
Education (mean±SD)*	9.06±5.50	8.76±5.25	0.47
Family income (mean±SD)*	22.5±30.0	21.2±26.6	0.58
MADRS*	6.7±6.2	6.6±6.0	0.34
SRQ*	3.4±2.9	3.4±2.9	0.91

* Student t test for independent samples;

** chi-square test (with Yates correction or Fisher exact).

The study was approved by the Ethics Committee for Research of the Hospital de Clínicas de Porto Alegre. All subjects and/or their proxies signed an informed consent.

### Data analysis

Statistical analysis was performed using the *Statistical Package for the Social Sciences* (SPSS for Windows 14.0). Student’s t test was used for independent samples and chi-square test – with Yates correction or Fisher exact. A multivariate logistic regression model was used to identify independence of the variables’ effect upon the outcome mortality. Statistical significance was defined as p<0.05.

## Results

The global mortality rate observed over the 8-year period was 36.9% (90 participants) in relation to the baseline sample. The analysis of the deceased group showed a mean age of 73.46 years (SD=7.48), mean educational attainment in years of 8.52 (SD=5.30) and gender frequency of 63% women (N=57). Women were more frequent in the group, as well being older and less educated than those who composed the alive group (Table 2).

**Table 2:** Socio-demographic baseline data of sample according to outcome (vital status) during follow-up.

Variables	Follow-up vital status		
	Deceased (N = 90)	Alive (N = 156)	P Value
Age (mean±SD)*	73.47±7.48	69.36±5.97	0.001
Sex (N, %)**			
Female	57 (63.3%)	113 (72.4%)	
Male	33(36.7%)	43(27.6%)	0.139
Education (in years)* (mean±SD)	8.5±5.3	8.8±5.2	0.632
Marital status (N, %)**			
Married	38 (42.2%)	76 (48.7%)	
Widow(er)	37 (41.1%)	66 (42.3%)	
Divorced	6 (6.7%)	2 (1.3%)	
Single	9 (10.0%)	11 (7.1%)	0.139
Family income (minimum wages)* (mean±SD)	21.19±27.33	21.09± 26.18	0.978

* Student's t test - independent samples;

** chi-square test (with Yates correction or Fisher exact).

Among those who had deceased, 80% (N=72) were under some kind of medication, and 51% (N=46) reported some type of health problem, while only 39% (N=61) among the alive participants presented the same profile (Table 3). Frequency of functional incapacity was higher among the deceased, and general movement difficulty such as leaving or entering the house or moving about outside the home were the most prevalent, 19% (N=17).

**Table 3:** Medical, cognitive and psychiatric baseline data of sample according to outcome (vital status) during follow-up.

	Follow-up vital status		
Variables	Deceased	Alive	P value
Global independence**	71 (78.9%)	151 (96.8%)	0.001
(Yes %)			
Incapacity for: (N,%)**	5 (5.6%)	1 (0.6%)	0.017
Locomotion	8 (8.9%)	1 (0.6%)	0.001
Cooking	7 (7.8%)	0	0.001
Hygiene	17 (18.9%)	4 (2.6%)	0.001
Movement			
General health problems**	46 (51.1%)	61 (39.1%)	0.068
(Yes %)			
Use of medications**	18 (20.0%)	39 (25.0%)	0.641
(No %)	72 (80.0%)	116 (74.4%)	
(Yes %)			
Reported Diseases (N,%) **	11 (12.2%)	20 (12.8%)	0.891
Diabetes	9 (10.0%)	10 (6.5%)	0.324
Lung	35 (38.9%)	41 (26.3%)	0.041
Heart	7 (7.9%)	8 (5.2%)	0.404
Cancer			
Mini Mental State (mean±SD)*	24.3±4.46	25.9±2.80	0.001
MADRS (mean±SD)*	7.1±6.12	6.45±6.06	0.419
SRQ (mean±SD)*	3.7±2.9	3.3±2.9	0.205

* Student's t test for independent samples;

** chi-square test for association (Yates correction or Fisher exact).

The elderly who were alive showed higher MMSE scores than those who deceased over the period. The cognitive deficit, demonstrated by the application of the education-adjusted cutoffs to the Mini Mental State Examination, showed association to mortality in the univariate analysis (p=0.001). The mean SRQ score and the mean MADRS were similar among the deceased and alive groups (Table 3).

In the multivariate logistic regression analysis, functional capacity (OR=4.58; 95% CI 1.52–13.79, p=0.007) and age (OR=1.07; 95% CI 1.02 – 1.12, p=0.003) showed significant association to the outcome – mortality – in the final equation with 244 cases having been included in the analysis (Table 4).

**Table 4:** Variables kept in the final model of the logistic regression: prediction of global mortality.

Variable	B	Wald	P value	OR (CI 95% Lower-Upper)
Independence*	1.522	7.331	0.007	4.58 (1.52-13.79)
Age	0.068	8.892	0.003	1.07 (1.02-1.12)
Sex**	0.582	3.188	0.074	1.79 (0.94-3.39)
Education	0.004	0.015	0.903	1.01 (0.95-1.64)
MMSE	-0.083	2.670	0.102	0.92 (0.83-1.02)
SRQ	0.046	0.790	0.374	1.05 (0.95-1.16)
Constant	-3.845	2.933	--	--

OR: odds ratio; CI 95%: confidence interval 95% lower and upper limits;

* Any functional incapacity is the reference;

** Male is the reference.

## Discussion

The present study was developed to evaluate the association of general psychiatric and depressive symptoms with mortality during the 8 years of follow-up in a community sample of elderly in Southern Brazil. The global mortality was 36.9%. In our sample, presence of general psychiatric or depressive symptoms was not associated to mortality.

Mortality rates varied greatly among studies, and were dependent on duration of follow-up, age bracket, cognitive impairment, and other factors, ranging from 10% to 87%.^27,28^

Stressing the characteristics of this sample, composed of very healthy and independent community elderly at baseline, is of importance because this could be the basis for lower levels of psychiatric disorders at some point in time in our their lives. The tools used were scales for general, non-psychotic, psychiatric or depressive symptoms which are not directly related to diagnosis (major depression or other psychiatric disorder). However, even if other methods for diagnosis might have been used to ascertain psychiatric disorder, the great variability observed among the available diagnostic systems supports diagnostic heterogeneity. These aspects may interfere in the relationship of presence of psychiatric diagnosis and mortality occurrence, and/or other similar magnitude outcomes.

In our study, cognitive decline was not associated to mortality. In contrast, it is very frequent to find studies demonstrating a direct association between cognitive decline and higher mortality.^29^

Age was associated to mortality in the multivariate analysis and this result was also found for this point in our cohort study. The selection criteria for the baseline sample – participants who fulfilled criteria for the study of healthy aging – allows for the presence of individuals who will reach exceptional ages, i.e., the oldest old, during forthcoming assessments. In these subjects, the association of traditional risk factors, such as age, with all-causes of mortality will change. Age was one of the risk factors for mortality in younger old people, but in the old old, predictors (age, sex, disability, self-reported health) of mortality have changed over time, and their predictive effects have eventually reduced.^30^ It has been reported that the exponential relationship of age with morbidity and mortality for people aged 65–84 years does not persist in those aged 90 years or more.^31^

We also observed the predictive effect of functional capacity (independence) upon mortality. Any degree of functional incapacity was a risk factor for mortality. In Brazil, a previous study had observed similar data.^32^

Another important aspect in the present study was the origin of the sample. The concept of catchment area is a worldwide notion. The population in the present case is composed of middle and higher classes from the city of Porto Alegre, which explains the higher educational level and average family income of the sample. It reflects the better general conditions and the social and medical assistance received by this group, which is protective against many of the well known mortality risk factors for the elderly.
